# Supplementary material for: The Peptide AWRK6 Alleviates Lipid Accumulation in Hepatocytes by Inhibiting miR-5100 Targeting G6PC
Source: Int J Mol Sci. 2023 Nov 9;24(22):16141. doi: 10.3390/ijms242216141 (PMC10671387; doi:10.3390/ijms242216141)
Supplement: Supplementary file 1 [file ijms-24-16141-s001.zip › ijms-2663458-supplementary.pdf]

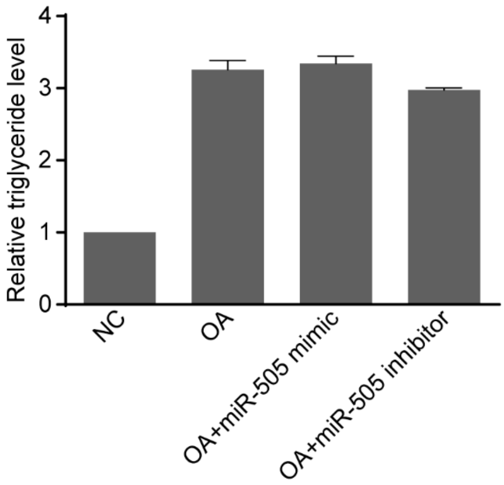

Figure S1. Triglyceride level was not altered by miR-505. The HepG2 cells were treated with oleic acid for 24 h, then transfected with miR-505 mimics and inhibitors. The triglyceride level was analyzed using Triglyceride Colorimetric Kit.
